# A multicenter randomized study: safety of an optimized accelerated house dust mite immunotherapy for patients with allergic rhinitis in China (PERFECT study)

**DOI:** 10.3389/fimmu.2026.1751162

**Published:** 2026-04-10

**Authors:** Guangyao He, Qingxiang Meng, Xuan Yuan, Yongzhen Liu, Qinhui Fu, Yongjing Lai, Mao Xie, Zhan Liu, Junyi Zhang, Hua Zhang, Fengjun Wang, Shumin Xie, Weihong Jiang, Zhihai Xie

**Affiliations:** 1 Department of Otolaryngology-Head and Neck Surgery, The First Affiliated Hospital of Guangxi Medical University, Nanning, China; 2 Department of Otolaryngology-Head and Neck Surgery, Guangzhou First People’s Hospital, School of Medicine, South China University of Technology, Guangzhou, China; 3 Department of Otolaryngology-Head and Neck Surgery, Xiangya Hospital of Central South University, Changsha, China; 4 Key Laboratory of Major Diseases of Otolaryngology in Hunan Province, Changsha, China; 5 National Clinical Research Center for Geriatric Diseases, Xiangya Hospital of Central South University, Changsha, China

**Keywords:** accelerated dose build-up, adolescents, adults, house dust mites, one-strength, safety, subcutaneous immunotherapy

## Abstract

**Background:**

The one-strength injection regimen has increasingly become the mainstream paradigm for subcutaneous immunotherapy (SCIT). This study aimed to assess the safety and tolerability of an optimal accelerated scheme for SCIT using a house dust mite (HDM) product in adolescents and adults with allergic rhinitis (AR) or rhinoconjunctivitis (ARC), with or without asthma in China.

**Methods:**

In this multicenter, open-label trial, patients were randomized to either One-strength or Standard group. The One-strength scheme included 6 injections with one-strength (5,000 TU/mL) followed by 4 injections at the maximum tolerated dose, administered without dose reduction when initiating a new vial. In contrast, the Standard regimen involved 14 injections across three strengths (50, 500 and 5,000 TU/mL), followed by 4 maintenance injections administered according to the medication instruction. Safety outcomes were determined by assessing Adverse Drug Reactions (ADRs), while tolerability was evaluated using a 5-point Likert scale.

**Results:**

Overall, 211 patients were randomized, with 108 assigned to the One-strength group, and 103 to the Standard group. Among all participants, 57.3% reported ADRs, of which 95.3% were local reactions. Systemic ADRs were observed in 7.4% of patients in the One-strength group and 8.7% in the Standard group, with no significant difference between the two groups (p=0.7233). All ADRs were classified as WAO grade 1 or 2, with no grade ≥3. In both adolescent and adult subgroups, the incidence of systemic ADRs remained comparable between the two arms (p > 0.05). However, in adults, only the incidence of local ADRs in the One-strength group was significantly higher than that in the Standard group (p=0.0004). Tolerability was rated as “good” or “very good” by approximately 84% of patients and investigators in the One-strength group and 70% of the Standard group (p=0.0131 for patient rating; p=0.0142 for investigator rating).

**Conclusions:**

The One-strength scheme, consisting of 6 injections and no dose reduction when starting a new pack during the maintenance phase, is as safe and tolerable as standard SCIT regimen in adolescents and adults in China.

**Clinical Trial Registration:**

www.chictr.org.cn, identifier ChiCTR2200060194.

## Introduction

Allergic rhinitis (AR) and asthma are highly prevalent airway allergic diseases, characterized by an inflammatory response mediated by immunoglobulin E (IgE) in allergen-sensitized individuals (1). The global prevalence of AR in adults ranges from 10% to 30% (2), while surveys in China have documented a self-reported rate of 34.3% (3). Asthma is a chronic and heterogeneous respiratory disease, with an estimated global prevalence of 4.3% (4). AR is widely regarded as a precursor to asthma, with shared genetic, microbial, and environmental determinants potentially contributing to the progression from AR to asthma (5). The house dust mites (HDM) *Dermatophagoides farinae (Der. f.)* and *Dermatophagoides pteronyssinus (Der. p.)*, represent the dominant perennial aeroallergens responsible for persistent symptoms in individuals with AR and allergic asthma (AA) in China (6, 7). A cross-sectional survey of Chinese patients with asthma and/or rhinitis showed that the prevalence of skin test positivity was 59% for *Der. f.* and 57.6% for *Der. p (*8*).*, with other studies reporting that 83.7%~99.86% of patients were simultaneously sensitized to both mites (9, 10). Overall, HDM remains the most clinically relevant indoor allergen implicated in AR and AA worldwide (11).

Allergen immunotherapy (AIT) has been a cornerstone treatment for allergic diseases caused by inhalant allergens for many decades. It is the only treatment modality that targets the underlying immunological mechanisms of allergic diseases and has the potential to prevent the progression of allergic rhinitis to asthma (7, 12). Nevertheless, adherence challenges persist, substantially influencing the overall effectiveness of AIT (13). Inconvenience due to the frequency of injections in the dose build-up and the occurrence of systemic reactions are common reasons for nonadherence and treatment discontinuation (14, 15). Moreover, according to the instructions for Novo Helisen^®^ Depot (NHD) — the sole approved dual-mite allergen preparation in China — a dose reduction is required when switching to a new vial during the maintenance phase, which may further negatively impact adherence.

To enhance patient adherence, various accelerated dose build-up protocols have been explored, including the previously reported rush and cluster immunotherapy regimens. These approaches are designed to expedite transition into the maintenance phase by administering multiple injections per day to improve patient convenience. Although these regimens have been introduced to reduce the frequency of clinic visits, they are often accompanied by greater injection-related anxiety and an elevated incidence of systemic reactions due to their intensive dosing design (16). Additionally, the multi-injection structure of these therapies presents challenges in determining the adverse event associated with each injection, complicating dose adjustments.

In recent years, multiple prospective controlled studies have evaluated the safety and tolerability of one-strength dose build-up modality for SCIT in patients with AR (17–24). These investigations have included 3-injection one-strength protocol with allergoid preparation of grass pollen, as well as 6-injection one-strength regimen using native HDM extracts. Collectively, these studies highlight that one-strength accelerated build-up scheme has emerged as a global mainstream trend, with strong potential to reshape future SCIT practice. In contrast to rush and cluster regimens, one-strength protocol truly reduces the total number of injections and enables more comprehensive assessment of adverse reactions associated with each administration. However, comprehensive safety data integrating the one-strength scheme in up-dosing phase with no dose reduction when switching vials during the maintenance phase remain scarce — particularly as no relevant data are available for Chinese adolescent or adult populations.

Therefore, the present multicenter, oPEn label, Randomized study aims to further investigate the saFEty of an optimal aCcelerated scheme for immunoTherapy in adolescent and adult patients with moderate to severe allergic rhinitis or rhinoconjunctivitis with or without asthma in China (PERFECT study). Should the results demonstrate that this regimen is both safe and well-tolerated, it could offer a more convenient, patient-friendly alternative to conventional AIT scheme, ultimately improving patient adherence and clinical outcomes.

## Methods

### Study design and participants

This was a multicenter, open-label, randomized study conducted between July 2022 and April 2024 at three hospitals in China.

Eligible participants were individuals aged 12 to 65 years diagnosed with moderate to severe AR or ARC according to ARIA (25), with or without asthma, induced by HDM allergens. To be eligible, participants were required to have experienced symptoms for at least one year and to have confirmed sensitization to Der. f. and/or Der. p. through a positive skin prick test (SPT) and/or elevated specific IgE levels (≥ 0.70 kU/L). Participants with asthma were required to have well-controlled symptoms as defined by the Global Initiative for Asthma (GINA) guidelines (26).

Key exclusion criteria included any history of systemic reactions or anaphylaxis following AIT, prior HDM-specific AIT within the past five years, or recent use of any other form of AIT. Individuals with clinically significant autoimmune diseases, immune deficiencies, or severe medical conditions that could compromise safety or adherence to the study protocol were excluded. Women who were pregnant or planning to become pregnant during the study, or those without reliable contraception, were also excluded. Additionally, participants with β-blocker use, or contraindications to epinephrine use (e.g., uncontrolled hypertension or symptomatic coronary artery disease) were deemed ineligible.

This study has been approved by the ethical committees of all participating hospitals and performed in line with the principles of the Declaration of Helsinki. Written informed consent to participate in the study was obtained from each patient, patient’s parent or guardian respectively before any study-related activities were performed. This study was registered at www.chictr.org.cn under the identifier ChiCTR2200060194.

### Interventions

Novo-Helisen^®^ Depot [NHD (*Der. p.*: *Der. f.* 50:50% by volume) (manufactured by Allergopharma GmbH & Co. KG, Reinbek, Germany), an aluminum hydroxide-adsorbed house dust mite SCIT product, was used for all patients.

**Figure 1:**
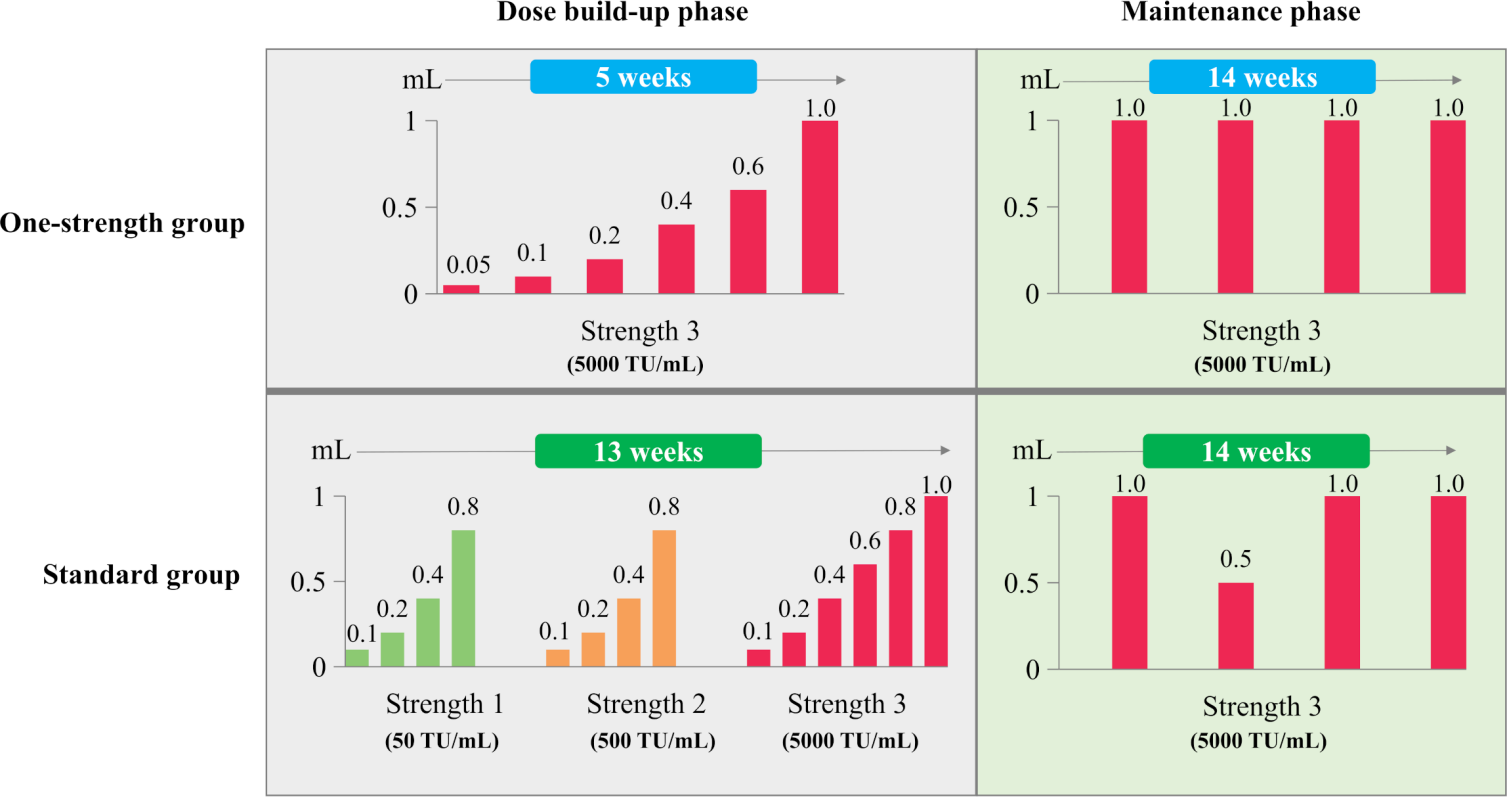
Dosage regimen during SCIT with the native house dust mite product in the One-strength or Standard group.

Patients were randomized 1:1 to the One-strength or Standard group. Randomization was performed block-wise at each site in a 1:1 ratio, with patients stratified into the respective age groups according to their age at the day of their enrolment. Patients in the One-strength group received 6 injections with build-up doses of strength 3 (5000 TU/mL) in a weekly interval over a period of 5 weeks. After completing the dose build-up phase, the maintenance dose was administered initially after a 2-week interval and subsequently every 4 weeks. Patients in the Standard group received a total of 14 injections for dose build-up phase in weekly intervals, of strength 1 (50 TU/mL), strength 2 (500 TU/mL), and strength 3 (5000 TU/mL) over a period of 13 weeks. Following the build-up phase, the maintenance dose was administered after a 2-week interval, followed by a subsequent dose at 4 weeks with the dose reduced by half (0.5 mL), and then every 4 weeks with the maintenance dose. (**Figure 1**). In both groups, patients were observed for at least 180 minutes following each injection to monitor for adverse reactions.

### Evaluation of safety and tolerability

Safety endpoints focused on the adverse drug reaction (ADR), which was defined as all untoward and unintended responses to the AIT related to any dose administered. A local ADR (LAR) was defined as adverse event (AE) related to AIT and occurring at the injection site. A systemic ADR (SAR) was an AE related to AIT and graded according to the WAO Subcutaneous Immunotherapy Systemic Reaction Grading System that is based on the organ systems involved and the severity of the reaction (27). An ADR was assessed as serious if the necessary treatment was given parenterally such as intramuscular and intravenous injections.

ADRs were coded using version 27.0 of the Medical Dictionary for Drug Regulatory Activities (MedDRA). All data on ADRs were listed together with the corresponding MedDRA primary System Organ Class (SOC) and Preferred Term (PT).

All ADRs were classified according to their intensity (severity) based on the following criteria: Mild: Transient symptoms, no interference with the patient’s daily activities. Moderate: Marked symptoms, moderate interference with the patient’s daily activities. Severe: Considerable interference with the patient’s daily activities.

Overall tolerability was assessed by the investigators and the patients using a 5-point Likert scale (1 = very bad; 5 = very good) (28).

Moreover, changes in laboratory values (hematology, clinical chemistry, and urinalysis) measured before and after the treatment phase, changes in vital signs and lung function measured before and after the treatment phase were documented.

### Statistical analysis

Statistical analyses were performed using SAS software (version 9.4). Descriptive statistics were used to summarize demographic and baseline characteristics. Continuous variables were expressed as means and standard deviations (SD) or medians and interquartile ranges (IQR), depending on data distribution, and categorical variables as frequencies and percentages. Group comparisons were conducted using the two-sided van Elteren-Test (stratified by age group) for continuous variables, and the two-sided Mantel-Haenszel test for categorical variables. For subgroup analysis, group comparisons were conducted using the Mann-Whitney U test for continuous variables, and the Fisher’s exact test for categorical variables. As the study was exploratory, no formal sample size calculation was performed, but a total of 140 participants (70 per group, with 35 adolescents and 35 adults) were planned to ensure a 95% probability of detecting an ADR incidence rate of 8.6%. To account for potential dropout, 200 participants were recruited. ADRs, SARs, and severe allergic reactions (graded by the WAO systemic reaction grading system) were compared between groups using appropriate exploratory tests. Laboratory values and vital signs were summarized with means ± SD, 95% CI, medians, and ranges. Tolerability results were expressed as frequencies and percentages, with appropriate exploratory tests used to compare group differences.

## Results

### Basic demographic and characteristics

**Figure 2:**
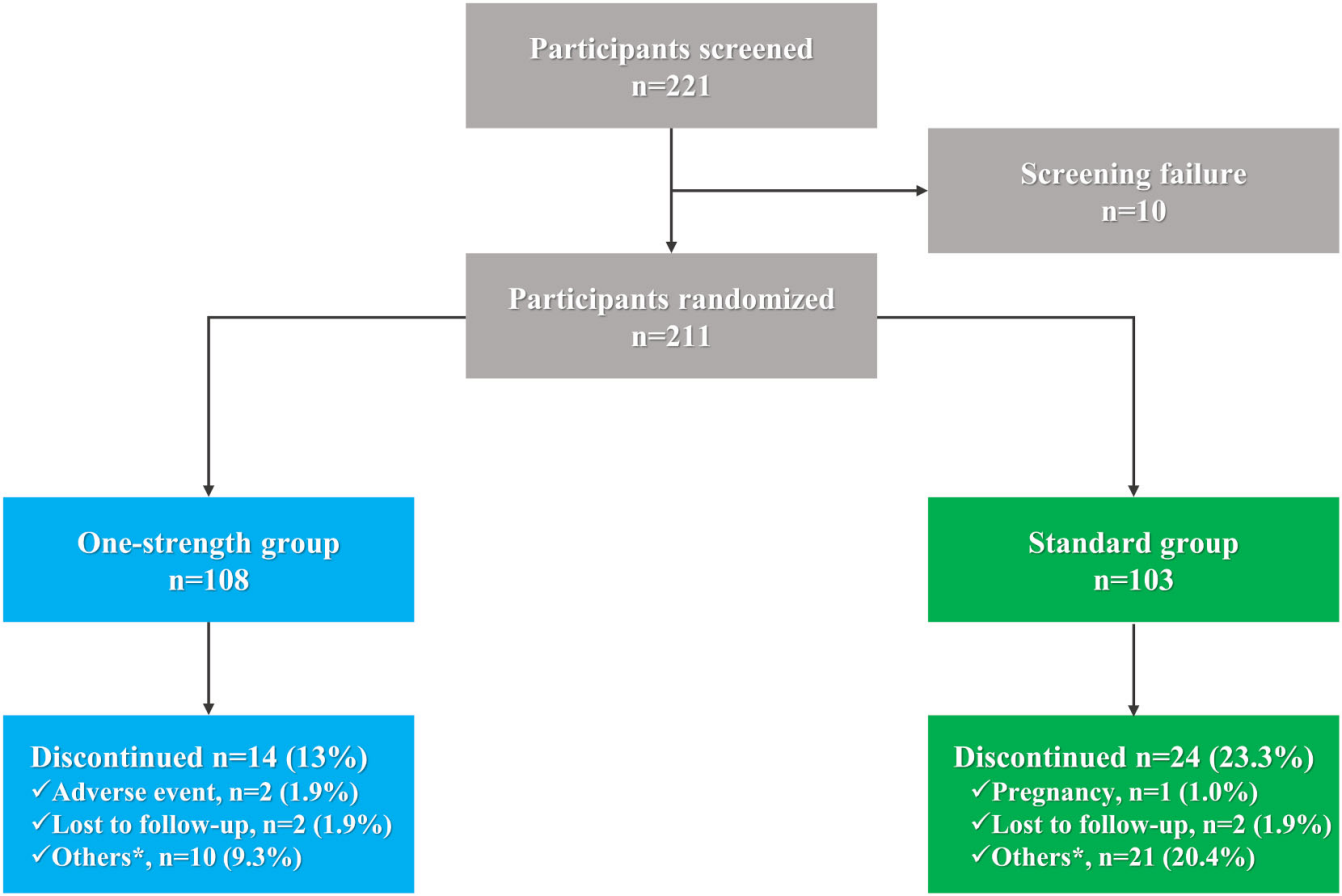
Patient flowchart. *, poor compliance, the patient requested withdrawal, COVID-19 pandemic restriction.

A total of 211 participants were enrolled and randomized, with 108 patients assigned to the One-strength group and 103 to the Standard group (**Figure 2**). All participants received at least one SCIT injection and were included in the safety analysis set (SAF). Demographic and baseline characteristics were comparable between groups, except for the presence of asthma, which differed significantly (2 (1.9%) vs.12 (11.7%) patients, p = 0.0043) (**Table 1**).

**Table 1:** Baseline demographic and clinical characteristics of patients in the safety set.

	Total			Adolescent (12–17 Years)			Adult (≥18 Years)		
Variable	One-strength group (n=108)	Standard group (n=103)	*P* value	One-strength group (n=54)	Standard group (n=49)	*P* value	One-strength group (n=54)	Standard group (n=54)	*P* value
Age, years			0.0617^1^			0.9946^2^			0.0101^2^
Mean (SD)	19.8(7.52)	22.8(11.16)		14.1(1.37)	14.1(1.60)		25.6(6.69)	30.7(10.18)	
Median (range)	17.5(12-47)	18.0(12-55)		14.0(12-17)	14.0(12-17)		24.5(18-47)	29.5(18-55)	
Sex, n (%)			0.1856^3^			0.0760^4^			1.0000^4^
Male	65(60.2)	71(68.9)		35(64.8)	40(81.6)		30(55.6)	31(57.4)	
Female	43(39.8)	32(31.1)		19(35.2)	9(18.4)		24(44.4)	23(42.6)	
BMI (kg*/*m^2^)	21.30(3.457)	21.50(3.806)	0.9653^1^	19.93(3.235)	20.64(4.431)	0.6944^2^	22.68(3.134)	22.29(2.963)	0.7470^2^
AR duration, years	7.5(4.69)	8.9(5.75)	0.1129^1^	6.8(3.61)	7.0(3.76)	0.7629^2^	8.2(5.51)	10.6(6.69)	0.0558^2^
Concomitant asthma, n (%)			0.0043^3^			0.3445^4^			0.0161^4^
Yes	2(1.9)	12(11.7)		1(1.9)	3(6.1)		1(1.9)	9(16.7)	
No	106(98.1)	91(88.3)		53(98.1)	46(93.9)		53(98.1)	45(83.3)	
FEV_1_ (% predicted)	87.49(12.616)	89.80(14.978)	0.2379^1^	84.62(7.942)	89.01(10.219)	0.0423^2^	90.35(15.541)	90.52(18.332)	0.7400^2^
sIgE									
Der p.	24.600(0.13-106)	25.400(0.39-100)	0.8271^1^	31.700(0.65-106)	44.500(0.93-100)	0.7232^2^	16.550(0.13-100)	12.600(0.39-100)	0.9681^2^
Der f.	28.100(0.59-100)	27.300(0.7-101)	0.5369^1^	36.800(0.59-100)	55.800(0.77-100)	0.3559^2^	17.700(0.72-100)	15.850(0.7-101)	0.9706^2^
Treatment duration									
Mean (SD)	142.9(42.43)	201.0(69.72)		140.9(44.14)	213.8(59.14)		145.0(40.97)	189.4(76.79)	
Median (range)	147.0(1-216)	220.0(9-320)		148.5(1 -203)	226.0(31-298)		145.5(8-216)	206.5(9-320)	
Achieved maintenance dose without dose adjustment, n (%)	83(76.9)	63(61.2)	0.0138^3^	42(77.8)	30(61.2)	0.0862^4^	41(75.9)	33(61.1)	0.1464^4^

Data are presented as mean (SD) or median (range). AR, Allergic Rhinitis; SD, Standard Deviation; BMI, Body Mass Index; FEV_1_, Forced Expiratory Volume in the first second; *Der p.*, *Dermatophagoides pteronyssinus*; *Der f., Dermatophagoides farinae*.

^1^p-value from the two-sided van Elteren-Test accounted for age group comparing both treatment groups.

^2^p-value from the two-sided Mann Whitney U-test comparing both treatment groups.

^3^p-value from the two-sided Mantel-Haenszel comparing both treatment groups.

^4^p-value from the Fisher’s Exact test comparing both treatment groups.

**Table 1** shows the distribution of patients by age group. A total of 103 adolescents were included, with 54 randomized to the One-strength and 49 to the Standard regimen. Of 108 adult patients, 54 were randomized to each treatment group. Adolescents of the Standard group showed a significantly higher FEV_1_ (p=0.0423), while adults in the Standard group were significantly older (p=0.0101) and suffered more often from asthma (p=0.0161) compared to the One-strength group. The remaining baseline demographic characteristics were not significantly different.

For the total population, the median treatment duration was 147.0 days in the One-strength and 220.0 days in the Standard group. Among the SAF, 76.9% of patients in the One-strength group and 61.2% of patients in the Standard group successfully reached the maintenance phase without dose adjustment (**Table 1**).

### Safety

The frequency of patients with at least one ADR was also higher in the One-strength group (69.4%) compared to the Standard group (45.6%) (p = 0.0005). Over 95% of ADRs in both groups were classified as mild (494/501 in the One-strength group vs. 361/378 in the Standard group), and none was categorized as severe. There was no significant difference in the number of patients with SARs between the groups (7.4% in the One-strength group vs. 8.7% in the Standard group, p = 0.7233) (**Table 2**).

**Table 2:** Number and proportion of patients in the safety set experiencing AEs.

	One-strength group (n=108)		Standard group (n=103)		*P* value
	Patients, n (%)	Events, n	Patients, n (%)	Events, n	
ADRs	75(69.4)	501	47(45.6)	378	0.0005
Dose build‑up phase	66(61.1)	379	43(41.7)	314	0.0050
Maintenance phase	36(33.3)	122	16(15.5)	64	0.0028
ADRs severity					
Mild	75(69.4)	494	45(43.7)	361	0.0002
Moderate	2(1.9)	7	5(4.9)	17	0.2709
Severe	0(0)	0	0(0)	0	NE
LARs	71(65.7)	486	41(39.8)	352	0.0002
Dose build‑up phase	62(57.4)	364	38(36.9)	293	0.0029
Maintenance phase	36(33.3)	122	13(12.6)	59	0.0004
SARs	8(7.4)	15	9(8.7)	21	0.7233
Dose build‑up phase	8(7.4)	15	7(6.8)	16	0.8632
Maintenance phase	0(0)	0	3(2.9)	5	0.1146
SARs -WAO grade					
WAO grade 1	7(6.5)	11	5(4.9)	7	0.6108
WAO grade 2	1(0.9)	4	5(4.9)	14	0.1122
ADRs leading to discontinuation	2(1.9)	3	0(0)	0	0.4979
Dose build‑up phase	2(1.9)	3	0(0)	0	0.4979
Maintenance phase	0(0)	0	0(0)	0	NE
ADRs leading to dose reduction	7(6.5)	25	7(6.8)	18	0.9270
Dose build‑up phase	7(6.5)	22	6(5.8)	15	0.8433
Maintenance phase	1(0.9)	3	2(1.9)	3	0.6144
Serious ADRs	5(4.6)	13	7(6.8)	22	0.4980
Dose build‑up phase	5(4.6)	13	5(4.9)	17	0.9389
Maintenance phase	0(0)	0	3(2.9)	5	0.1146

ADR, Adverse Drug Reaction; NE, not evaluable; AEs, Adverse Events; LARs, Local Adverse Reactions; SARs, Systemic Allergic Reactions. p-value from the Two-sided Mantel-Haenszel or Fisher’s Exact test comparing both treatment groups.

**Figure 3:**
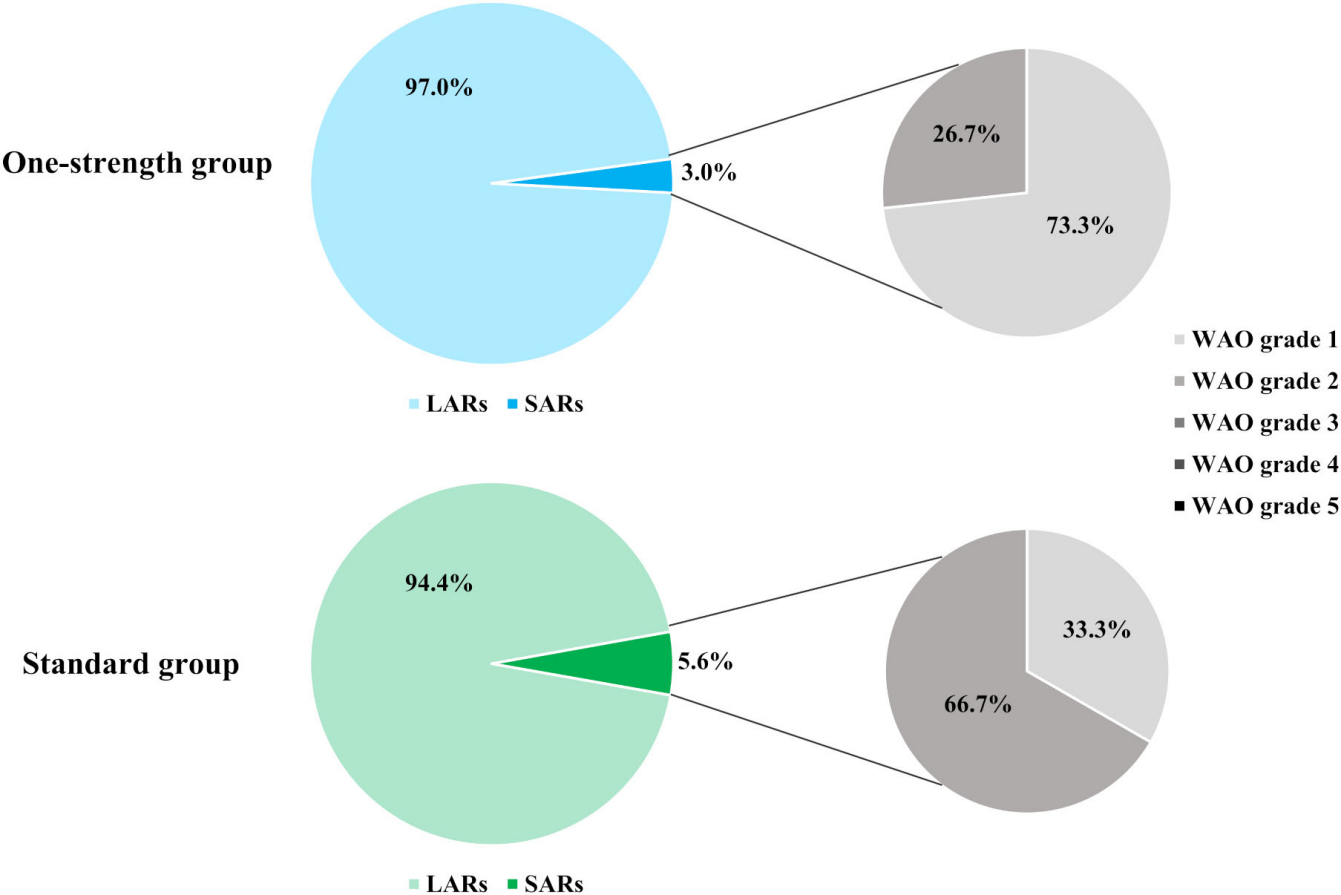
Distribution of local and systemic ADRs, and WAO grading of SARs. WAO, World Allergy Organization; ADR, adverse drug reaction; LAR, local adverse drug reaction; SAR, systemic adverse drug reaction.

Overall, 95.3% (838 out of 879) of all ADRs were LARs, with a comparable distribution across both treatment groups. In the One-strength group, SARs accounted for 3% of reported ADRs, and 73.3% (11/15) of these were classified as WAO grade 1. In contrast, 5.6% of ADRs in the Standard group were systemic, with 33.3% (7/21) categorized as WAO grade 1 (**Figure 3**). There were no significant differences in the number of patients experiencing SARs of WAO grade 1 or 2 between the One-strength and Standard group (p=0.6108 and p=0.1122, respectively). No SAR of WAO grade 3 or higher was reported.

All serious ADRs were systemic and occurred in both groups: 5 patients of the One-strength group were affected by 13 events while 7 patients of the Standard group developed 22 events without a significant difference between the two groups (p=0.4980). All serious ADRs occurred during the dose build-up phase in the One-strength group, while 17 events in 5 patients occurring during the dose build-up phase and 5 events in 3 patients occurring during the maintenance phase in the standard group. There was no serious ADR after 24 hours in either group. Moreover, 1 patient of One-strength group was treated with adrenaline due to ADR, while there were 5 patients of the Standard group (**Supplementary Table 1**).

### Age-related ADRs comparison

**Figure 4:**
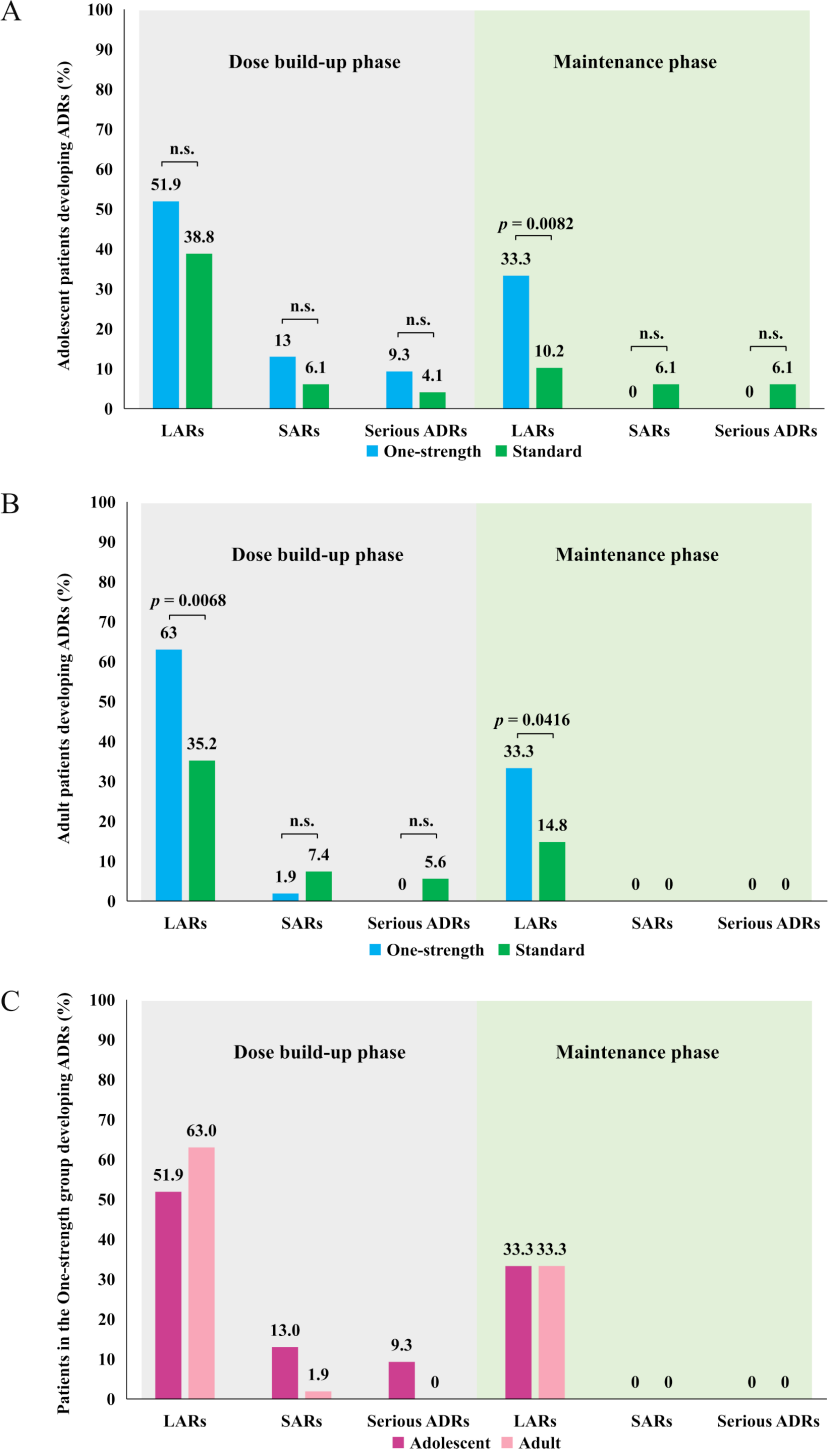
Incidence of local, systemic, and serious ADRs during the dose build-up and maintenance phases in adolescents **(A)** adults **(B)** and horizontal comparison between age cohorts in the One-strength group **(C)**. ADR, adverse drug reaction; LAR, local adverse drug reactions; SAR, systemic adverse drug reactions. n.s., not significant.

During the dose build-up phase, adolescents exhibited similar rates of local, systemic, and serious ADRs across both treatment groups (**Figure 4A**). In the maintenance phase, 33.3% of adolescents in the One-strength group reported LARs, significantly higher than the 10.2% observed in the Standard group (p=0.0082). No systemic or serious ADRs were reported in the One-strength group, and differences between the two groups were not statistically significant.

Among adult participants, the One-strength group showed LAR rates of 63.0% during build-up and 33.3% during maintenance, both significantly higher than the 35.2% and 14.8% observed in the Standard group (p=0.0068 and p=0.0416, respectively). Consistent with the adolescent findings, systemic and serious ADR rates did not differ significantly between groups during the build-up phase, and no such events occurred in either group during maintenance phase (**Figure 4B**). Types of ADRs with the corresponding MedDRA SOC and PT were listed in **Table 3**.

**Table 3:** Type of ADRs with the corresponding MedDRA primary system organ class and preferred term.

	Total			Adolescent (12–17 Years)			Adult (≥18 Years)		
	One-strength group (n=108)	Standard group (n=103)	*P value*	One-strength group (n=54)	Standard group (n=49)	P value	One-strength group (n=54)	Standard group (n=54)	*P value*
	Patients, n (%)	Patients, n (%)		Patients, n (%)	Patients, n (%)		Patients, n (%)	Patients, n (%)	
In total	75(69.4)	47(45.6)	0.0005	36(66.7)	26(53.1)	0.2264	39(72.2)	21(38.9)	0.0009
Respiratory, Thoracic, and Mediastinal Disorders	3(2.8)	3(2.9)	1.0000	3(5.6)	2(4.1)	1.0000	0(0)	1(1.9)	1.0000
Dyspnea	0(0)	2(1.9)		0(0)	2(4.1)		0(0)	0(0)	
Cough	2(1.9)	1(1.0)		2(3.7)	0(0)		0(0)	1(1.9)	
Throat Irritation	1(0.9)	0(0)		1(1.9)	0(0)		0(0)	0(0)	
Skin and Subcutaneous Tissue Disorders	5(4.6)	11(10.7)	0.1211	5(9.3)	6(12.2)	0.7531	0(0)	5(9.3)	0.0567
Erythema	2(1.9)	4(3.9)		2(3.7)	1(2.0)		0(0)	3(5.6)	
Hyperhidrosis	0(0)	1(1.0)		0(0)	0(0)		0(0)	1(1.9)	
Rash	2(1.9)	4(3.9)		2(3.7)	3(6.1)		0(0)	1(1.9)	
Eczema	0(0)	1(1.0)		0(0)	1(2.0)		0(0)	0(0)	
Urticaria	0(0)	1(1.0)		0(0)	0(0)		0(0)	1(1.9)	
Pruritus	4(3.7)	6(5.8)		4(7.4)	3(6.1)		0(0)	3(5.6)	
General Disorders and Administration Site Conditions	72(66.7)	42(40.8)	0.0002	33(61.1)	22(44.9)	0.1164	39(72.2)	20(37.0)	0.0004
Fever	0(0)	1(1.0)		0(0)	1(2.0)		0(0)	0(0)	
Chest Discomfort	3(2.8)	1(1.0)		2(3.7)	0(0)		1(1.9)	1(1.9)	
Injection Site Erythema	63(58.3)	32(31.1)		29(53.7)	16(32.7)		34(63.0)	16(29.6)	
Injection Site Rash	2(1.9)	4(3.9)		1(1.9)	3(6.1)		1(1.9)	1(1.9)	
Injection Site Pain	4(3.7)	0(0)		1(1.9)	0(0)		3(5.6)	0(0)	
Injection Site Swelling	62(57.4)	35(34.0)		27(50.0)	17(34.7)		35(64.8)	18(33.3)	
Injection Site Pruritus	64(59.3)	33(32.0)		28(51.9)	15(30.6)		36(66.7)	18(33.3)	
Vascular Diseases	0(0)	2(1.9)	0.2371	0(0)	0(0)	NE	0(0)	2(3.7)	0.4953
Facial Pallor	0(0)	1(1.0)		0(0)	0(0)		0(0)	1(1.9)	
Facial Flushing	0(0)	1(1.0)		0(0)	0(0)		0(0)	1(1.9)	
Central Nervous System Diseases	1(0.9)	0(0)	1.0000	0(0)	0(0)	NE	1(1.9)	0(0)	1.0000
Drowsiness	1(0.9)	0(0)		0(0)	0(0)		1(1.9)	0(0)	

NE, not evaluable; p-value from the Two-sided Mantel-Haenszel or Fisher’s Exact test comparing both treatment groups.

Focusing solely on participants receiving the One-strength formulation, age-related patterns unravel. During build-up, LARs were reported in 51.9% of adolescents compared with 63.0% of adults, while systemic and serious ADR rates were 13% and 9.3% for adolescents, and 1.9% and 0% for adults. In the maintenance phase, LAR rates became similar between the two age groups, and no systemic or serious ADRs were reported in either group (**Figure 4C**).

### Systemic adverse reactions distribution

**Figure 5:**
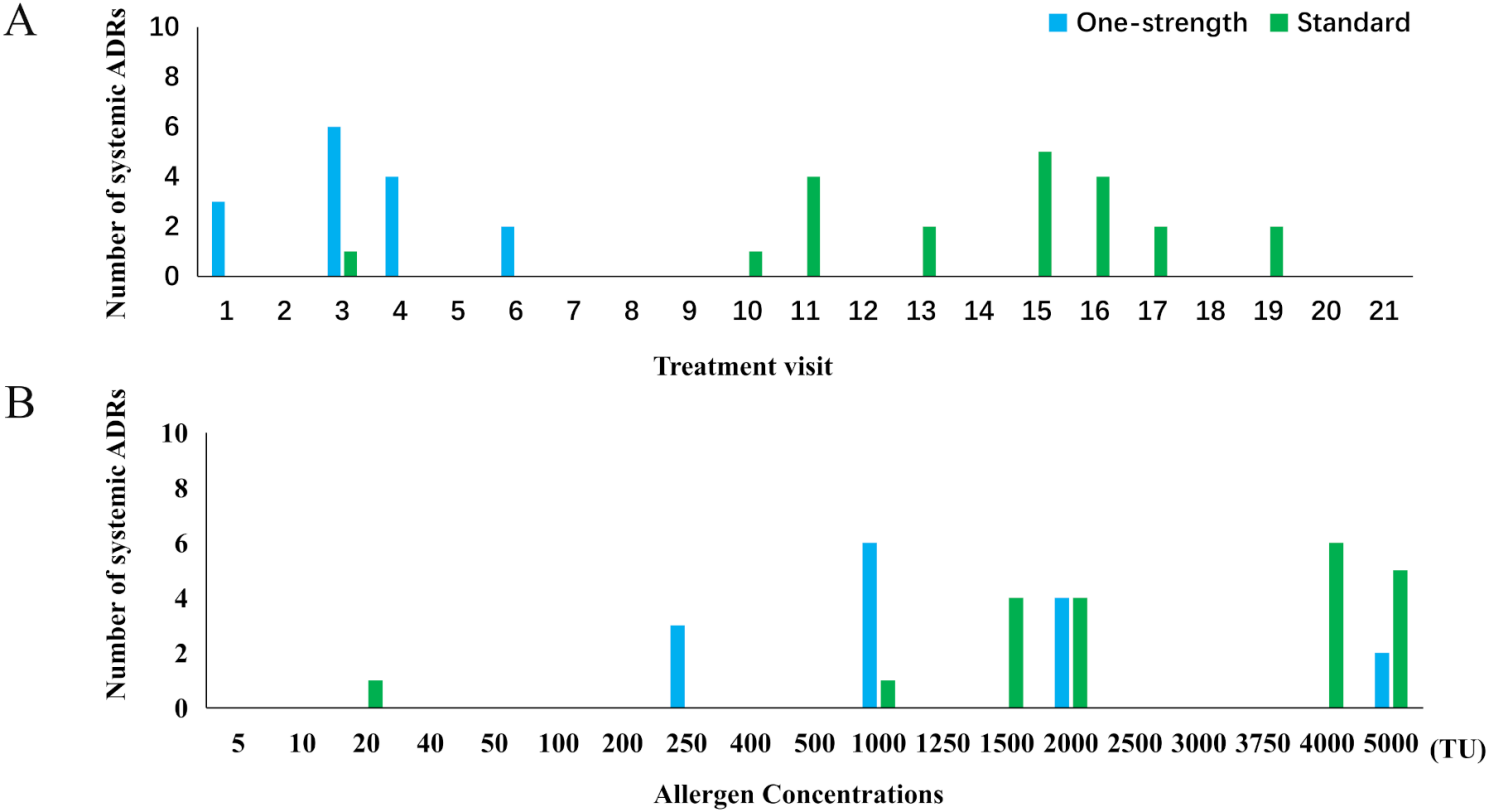
Distribution of the number of systemic ADRs by treatment visit **(A)** and by allergen concentrations **(B)**. ADR, adverse drug reaction.

SARs serve as a critical indicator of the safety profile of SCIT, with their occurrence closely associated with both the treatment phase and the administered dose. Analysis based on treatment visits (**Figure 5A**) demonstrated that all SARs within the One-strength group were confined to the build-up phase, peaking at the third injection (n=6). Conversely, SARs in the Standard group primarily manifested during visits 11, 15, and 16 (n=4, 5, and 4, resp.), corresponding to the early maintenance period.

Dose-based evaluation (**Figure 5B**) indicated that SARs in the One-strength group predominantly occurred at 1000 TU (0.2 mL of vial 3; 40%) and 2000 TU (0.4 mL of vial 3; 26.7%), whereas in the Standard group, SARs were mainly concentrated at 4000 TU (0.8 mL of vial 3; 28.6%) and 5000 TU (1.0 mL of vial 3; 23.8%).

### Tolerability and other safety parameters

**Figure 6:**
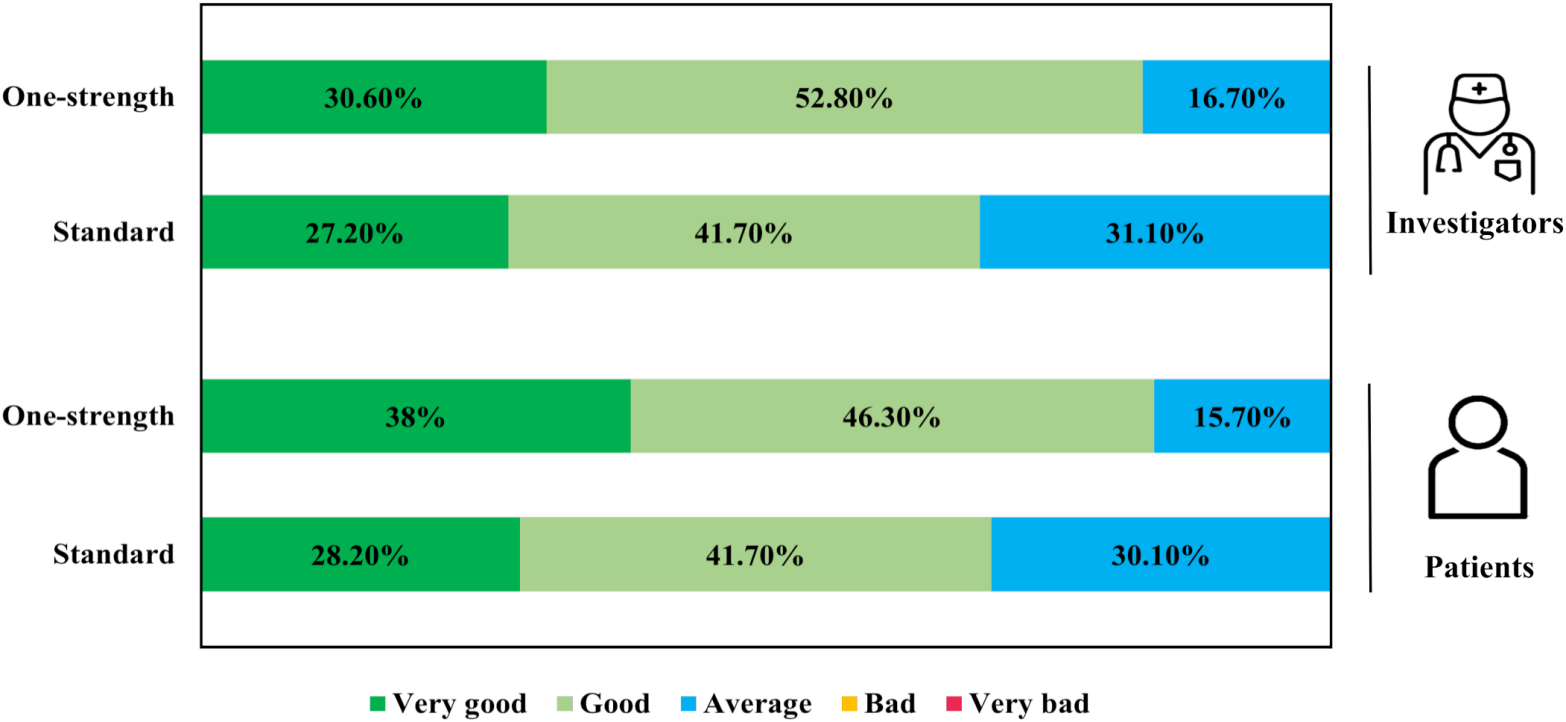
Assessment of overall tolerability by investigators and patients. Tolerability was evaluated using a 5-point Likert scale (very bad - bad - average - good - very good) at the last visit. (One-strength group, n=108; Standard group, n=103).

For the total study population, the One-strength regimen was rated as “very good” or “good” by 83.4% of investigators and 84.3% of patients, both notably greater than the corresponding proportions for the Standard regimen (p=0.0142 and p=0.0131, respectively; **Figure 6**). No clinically significant changes were observed in laboratory parameters, including clinical chemistry, hematology, or urinalysis. Additionally, there were no relevant changes in vital signs or lung function at the final visit.

## Discussion

The findings of this study demonstrate the safety and tolerability of the One-strength accelerated SCIT scheme compared to the Standard scheme in patients with AR, with or without asthma. These advantages are especially evident in the shorter time required to reach the maintenance dose, fewer injections, and the overall comparable safety profile. Such factors play a critical role in improving patient adherence to the recommended treatment duration of 3 years for AIT, which is necessary to achieve early therapeutic effects, progressive improvement over time, and long-term effects after termination of AIT in patients with allergic rhinitis and/or asthma (11, 12, 29).

Although the incidence of ADRs was significantly higher in the One-strength group than in the Standard group, most ADRs were mild and predominantly LARs. As previously reported, LARs are commonly observed during both the build-up and maintenance phases of AIT (30). In our study, the frequency of LARs was in agreement with previous findings, where 26-86% of patients receiving SCIT experienced LARs (31). Importantly, none of these LARs led to treatment discontinuation or dose reduction, which aligns with the outcomes of other studies conducted in adults (22).

The findings of this study reveal that SARs occurred in 7.4% of patients in the One-strength group and 8.7% in the Standard group, which is comparable to a recently published real-life study in China, which reported SARs for 9.61% of patients (32). Importantly, there was no indication of increased severity associated with the One-strength regimen, as about 73% of SARs were classified as WAO grade 1, compared with 33% in the Standard group. Notwithstanding, no events were classified as WAO grade 3 or higher. Therefore, the overall frequency and intensity of SARs were not considered a safety concern, nor did they suggest any need to modify the benefit-risk profile of the product, consistent with other studies (17, 22, 33). In total, 76.9% of patients in the One-strength group reached the maintenance dose without dose adjustments due to adverse events, compared to 61.2% in the Standard group. As noted in previous studies, most ADRs requiring dose reduction tend to occur during the dose build-up period (34). Only 3 ADRs resulted in treatment discontinuation in 2 patients in our study, suggesting that patients who completed the build-up phase were able to achieve the target dose concentration without major issues.

This study conducted a further analysis of the applicability of the One-strength scheme across different age groups. Adolescents demonstrated comparable safety outcomes to adults, which is particularly relevant as younger patients often exhibit lower adherence to long-term treatment regimens (35). By reducing the treatment burden through fewer injections and a shorter overall treatment duration, the One-strength scheme offers a practical solution to enhance adherence in this population, potentially contributing to better long-term outcomes. Both age groups benefited from the accelerated dosing approach. In adolescents, the median treatment duration was 148.5 days in the One-strength group compared with 226.0 days in the Standard group. Similarly, the median treatment duration in adults in the One-strength group was 145.5 days, as opposed to 206.5 days in the Standard group. These findings align with previous studies on accelerated SCIT schemes (23, 24). Reducing the time and number of injections required to reach the maintenance dose is particularly valuable in enhancing adherence among younger patients, who may find lengthy treatment durations challenging.

During the dose build-up phase, the one-strength regimen did not elevate the risk of local, systemic, or serious ADRs in adolescent populations. In adults, however, LARs occurred more frequently with the One-strength regimen compared with the Standard schedule, whereas the incidence of systemic or serious reactions remained unchanged. For the maintenance protocol that switched vials without dose reduction, only LAR rates increased in both age groups. These findings provide valuable insights for the clinical application of one-strength accelerated protocols. Greater focus should be placed on injection-site reactions, which may be mitigated via bilateral injections and the prompt application of ice packs post-injection.

Two studies from China and Europe have examined the application of the one-strength scheme for HDM SCIT in patients with AR. A comparative analysis of SARs incidence across these studies may provide additional perspectives. The TiME study, a European multicenter randomized controlled trial (RCT), included participants aged 12 to 65 years, whereas the FAST study, a Chinese multicenter RCT, enrolled children aged 5 to 14 years. The incidence of SARs across these three studies can be analyzed by two dimensions (**Supplementary Figure 1**). First, with respect to age groups, the proportion of patients experiencing SARs under the One-strength scheme was similar among children, adolescents, and adults, all at approximately 10%. Second, regarding ethnicity, adolescents receiving the one-strength scheme in the present study exhibited SAR frequencies comparable to those reported in the TiME study. However, adults in the European cohort exhibited a higher incidence of SARs than those in the Asian population. While the limited sample size may partially explain this discrepancy, variations in cultural factors and clinical practices could also influence ADR reporting rates. Consequently, further large-scale, multi-ethnic investigations are warranted to allow more comprehensive evaluation and validation of these findings.

Given that nearly all ADRs occurred during the build-up phases when administering Strength 3 also in the Standard dose build-up regimen, the feasibility of a One-strength scheme that bypasses Strengths 1 and 2 is supported. Notably, no SAR was observed in the One-strength group, whereas 5 SARs occurred in 3 patients in the Standard group during the maintenance phase. This suggests that the One-strength scheme, without dose reduction when transitioning to a new vial during the maintenance phase, is safe and well-tolerated. From the perspective of injection dosage, across both groups, a high incidence of SARs was observed with 2000 TU (0.4 mL of vial 3); thus, greater attention should be paid to this specific injection in clinical practice.

The overall tolerability of both dose schemes was rated as “very good” or “good” by the majority of investigators and patients, consistent with results from previous studies involving adults and children (17, 22, 36).

From a clinical perspective, the One-strength scheme addresses several key challenges associated with AIT. Under current usage instruction approved in China, the maintenance phase typically involve a 50% dose reduction when transitioning to a new vial, a procedure that is inconvenient for physicians and clinic staff. Additionally, the high-dose allergen concentrations used in AIT are specifically intended to induce immune tolerance (37). The faster attainment of the maintenance dose, together with fewer required injections, improves patient convenience and facilitates smoother treatment management for healthcare providers. This is particularly beneficial in regions like China, where adherence to long-term therapies is often hindered by the burden of frequent clinic visits and lengthy treatment durations. The comparable safety profile of the One-strength scheme relative to the Standard scheme further supports its value as a feasible alternative to conventional SCIT protocols. Given the rising prevalence of allergic diseases in China, implementing efficient and patient-friendly treatment regimens such as the One-strength scheme may positively influence public health outcomes by lowering barriers to adherence and improving overall patient outcomes.

This study has several notable strengths. In Europe, allergoid preparations are primarily used for SCIT, whereas only native allergen preparations are available in China. This study is the first randomized, controlled investigation of safety of a One-strength dose build-up scheme in adolescents and adults with HDM-allergic AR or ARC, with or without well-controlled asthma, in China. One limitation is the open-label design. Blinding was impossible given the completely different injection schedules and dosages. Although this could potentially influence the reporting of subjective symptoms, we mitigated this by evaluating and grading all adverse events using standardized criteria.

In conclusion, our findings demonstrate that the One-strength scheme with 6 injections plus no dose reduction when starting a new pack during the maintenance phase is as safe and tolerable as standard SCIT regimen in Chinese adolescents and adults. It also significantly reduces treatment duration and the number of injections needed to reach the maintenance dose, showing favorable tolerability across age groups and a safety profile comparable to conventional methods. This positions the One-strength scheme as a promising option for improving adherence and achieving better clinical outcomes in patients with HDM-allergic AR. Future research should explore its potential to promote sustained immunological tolerance and assess its cost-effectiveness.

## Data Availability

The original contributions presented in the study are included in the article/**Supplementary Material**. Further inquiries can be directed to the corresponding authors.

## Supplementary material

Supplementary Figure 1 Incidence of systemic ADRs with the one-strength scheme of the native HDM extract across FAST, TiME and this study in different age groups. ADR, adverse drug reaction.

## References

[1] Bjermer L Westman M Holmström M and Wickman MC . The complex pathophysiology of allergic rhinitis: scientific rationale for the development of an alternative treatment option. Allergy Asthma Clin Immunol. (2019) 15:24. doi: 10.1186/s13223-018-0314-1, PMID: 31015846 PMC6469109

[2] Passali D Cingi C Staffa P Passali F Muluk NB and Bellussi ML . The International Study of the Allergic Rhinitis Survey: outcomes from 4 geographical regions. Asia Pac Allergy. (2018) 8:e7. doi: 10.5415/apallergy.2018.8.e7, PMID: 29423374 PMC5796967

[3] Zhang Y Zhang L . Prevalence of allergic rhinitis in China. Allergy Asthma Immunol Res. (2014) 6:105–13. doi: 10.4168/aair.2014.6.2.105, PMID: 24587945 PMC3936037

[4] Loftus PA Wise SK . Epidemiology of asthma. Curr Opin Otolaryngol Head Neck Surg. (2016) 24:245–9. doi: 10.1097/MOO.0000000000000262, PMID: 26977741

[5] Saranz RJ Lozano A Lozano NA Alegre G and Ponzio MF . The roadmap from allergic rhinitis to asthma. Curr Treat Options Allergy. (2020) 7:110–23. doi: 10.1007/s40521-020-00245-z, PMID: 41940407

[6] Zhang Y Zhang L . Increasing prevalence of allergic rhinitis in China. Allergy Asthma Immunol Res. (2019) 11:156–69. doi: 10.4168/aair.2019.11.2.156, PMID: 30661309 PMC6340797

[7] Wang C Bao Y Chen J Chen X Cheng L Guo YS . Chinese guideline on allergen immunotherapy for allergic rhinitis: the 2022 update. Allergy Asthma Immunol Res. (2022) 14:604–52. doi: 10.4168/aair.2022.14.6.604, PMID: 36426395 PMC9709690

[8] Li J Sun B Huang Y Lin X Zhao D Tan G . A multicentre study assessing the prevalence of sensitizations in patients with asthma and/or rhinitis in China. Allergy. (2009) 64:1083–92. doi: 10.1111/j.1398-9995.2009.01967.x, PMID: 19210346

[9] He J Gao J Zhao Y and Chen S . Distributional characteristics analysis of allergens in patients with allergic rhinitis in southern fujian province, China. J Asthma Allergy. (2024) 17:477–89. doi: 10.2147/JAA.S453914, PMID: 38798279 PMC11128227

[10] Chen ZG Li YT Wang WH Tan KS Zheng R Yang LF . Distribution and determinants of dermatophagoides mites sensitization ofAllergic rhinitis and allergic asthma in China. Int Arch Allergy Immunol. (2019) 180:17–27. doi: 10.1159/000499409, PMID: 31104060

[11] Agache I Lau S Akdis CA Smolinska S Bonini M Cavkaytar O . EAACI Guidelines on Allergen Immunotherapy: House dust mite-driven allergic asthma. Allergy. (2019) 74:855–73. doi: 10.1111/all.13749, PMID: 31095767

[12] Roberts G Pfaar O Akdis CA Ansotegui IJ Durham SR Gerth van Wijk R . EAACI guidelines on allergen immunotherapy: allergic rhinoconjunctivitis. Allergy. (2018) 73:765–98. doi: 10.1111/all.13317, PMID: 28940458

[13] Mahesh PA Vedanthan PK Amrutha DH Giridhar BH Prabhakar AK . Factors associated with non-adherence to specific allergen immunotherapy in management of respiratory allergy. Indian J Chest Dis Allied Sci. (2010) 52:91–5. doi: 10.5005/ijcdas-52-2-91, PMID: 20578401

[14] Reisacher WR Visaya JM . Patient adherence to allergy immunotherapy. Curr Opin Otolaryngol Head Neck Surg. (2013) 21:256–62. doi: 10.1097/MOO.0b013e32835f8048, PMID: 23549440

[15] Silva D Pereira A Santos N and Plácido JL . Costs of treatment affect compliance to specific subcutaneous immunotherapy. Eur Ann Allergy Clin Immunol. (2014) 46:87–94. 24739128

[16] Winslow AW Turbyville JC Sublett JW Sublett JL and Pollard SJ . Comparison of systemic reactions in rush, cluster, and standard-build aeroallergen immunotherapy. Ann Allergy Asthma Immunol. (2016) 117:542–5. doi: 10.1016/j.anai.2016.09.005, PMID: 27788885

[17] Bovermann X Ricklefs I Vogelberg C Klimek L and Kopp MV . Accelerated dose escalation with 3 injections of an aluminum hydroxide-adsorbed allergoid preparation of 6 grasses is safe for children and adolescents with moderate to severe allergic rhinitis. Int Arch Allergy Immunol. (2021) 182:524–34. doi: 10.1159/000512561, PMID: 33503610

[18] Buczyłko K van der Werf JF Boot D van Ree R . Accelerated up-dosing of subcutaneous immunotherapy with a registered allergoid birch pollen preparation. Int Arch Allergy Immunol. (2017) 172:183–6. doi: 10.1159/000464103, PMID: 28380494

[19] Chaker AM Al-Kadah B Luther U Neumann U and Wagenmann M . An accelerated dose escalation with a grass pollen allergoid is safe and well-tolerated: a randomized open label phase II trial. Clin Transl Allergy. (2015) 6:4. doi: 10.1186/s13601-016-0093-z, PMID: 26839682 PMC4736162

[20] Horn A Fernández-Rivas M Wolf H Ghaussy N Møller Kruse T Koutromanou K and Wüstenberg E . Shortened up-dosing with 7 injections of subcutaneous allergy immunotherapy (Alutard SQ) is safe and well tolerated. J Investig Allergol Clin Immunol. (2021) 31:80–3. doi: 10.18176/jiaci.0561, PMID: 32573456

[21] Kopp MV Bovermann X and Klimek L . Accelerated dose escalation with three injections of an aluminum hydroxide-adsorbed allergoid preparation of six grasses is safe for patients with moderate to severe allergic rhinitis. Int Arch Allergy Immunol. (2020) 181:94–102. doi: 10.1159/000503684, PMID: 31865326 PMC7050673

[22] Zielen S Plückhahn K Akboga Y Rieker-Schwienbacher J Thieme U and Rosewich M . Fast up-dosing with a birch allergoid is safe and well tolerated in allergic rhinitis patients with or without asthma. Immunotherapy. (2019) 11:177–87. doi: 10.2217/imt-2018-0143, PMID: 30730274

[23] Jutel M Vogelberg C Duwensee K Troyke D Klimek L . One-strength dose escalation of house dust mite depot product for subcutaneous immunotherapy is safe and tolerable. Allergy. (2024) 80:807–16. 10.1111/all.16370 PMC11891436 39540587

[24] Zhi L Bai Y Liao W Chen G Gao T Wan X . The safety and tolerability of a one strength dose-escalation scheme for subcutaneous immunotherapy with a native house dust mite extract in Chinese children: A multicenter, randomized, open label clinical trial. Heliyon. (2024) 10:e29450. doi: 10.1016/j.heliyon.2024.e29450, PMID: 38655350 PMC11036000

[25] Brozek JL Bousquet J Baena-Cagnani CE Bonini S Canonica GW Casale TB . Allergic rhinitis and its Impact on asthma (ARIA) guidelines: 2010 revision. J. Allergy Clin. Immunol. (2010) 126(3):466–76. doi: 10.1016/j.jaci.2010.06.047, PMID: 20816182

[26] Bateman ED Hurd SS Barnes PJ Bousquet J Drazen JM FitzGerald JM . Global strategy for asthma management and prevention: GINA executive summary. Eur Respir J. (2008) 31:143–78. doi: 10.1183/09031936.00138707, PMID: 18166595

[27] Cox L Larenas-Linnemann D Lockey RF Passalacqua G . Speaking the same language: The World Allergy Organization Subcutaneous Immunotherapy Systemic Reaction Grading System. J Allergy Clin Immunol. (2010) 125(3):569–74. doi: 10.1016/j.jaci.2009.10.060, PMID: 20144472

[28] Likert R . A technique for the measurement of attitudes. Arch Psychol. (1932) 22:5–15.

[29] Halken S Larenas-Linnemann D Roberts G Calderón MA Angier E Pfaar O . EAACI guidelines on allergen immunotherapy: Prevention of allergy. Pediatr Allergy Immunol. (2017) 28:728–45. doi: 10.1111/pai.12807, PMID: 28902467

[30] Coop CA . Local reactions from subcutaneous allergen immunotherapy. Immunotherapy. (2013) 5:1339–45. doi: 10.2217/imt.13.143, PMID: 24283844

[31] James C Bernstein DI . Allergen immunotherapy: an updated review of safety. Curr Opin Allergy Clin Immunol. (2017) 17:55–9. doi: 10.1097/ACI.0000000000000335, PMID: 27906697 PMC5644500

[32] Xu Q Jia J Lin H Liang D Chen H Wang Y . Systemic reactions to house dust mite subcutaneous immunotherapy in patients with allergic rhinitis and/or asthma: A real-life, multi-center study. Allergy. (2024) 80:1506–8. 10.1111/all.16254 PMC12105055 39056450

[33] Tophof MA Hermanns A Adelt T Eberle P Gronke C Friedrichs F . Side effects during subcutaneous immunotherapy in children with allergic diseases. Pediatr Allergy Immunol. (2018) 29:267–74. doi: 10.1111/pai.12847, PMID: 29247543

[34] Di Bona D Magistà S Masciopinto L Lovecchio A Loiodice R Bilancia M . Safety and treatment compliance of subcutaneous immunotherapy: A 30-year retrospective study. Respir Med. (2020) 161:105843. doi: 10.1016/j.rmed.2019.105843, PMID: 31778936

[35] Vogelberg C Brüggenjürgen B Richter H and Jutel M . House dust mite immunotherapy in Germany: real-world adherence to a subcutaneous allergoid and a sublingual tablet. Allergo J Int. (2021) 30:183–91. doi: 10.1007/s40629-020-00155-1, PMID: 41940407

[36] Xiang L Liu F Zhi L Jiang W Liu C Xie H . Safety of semi-depot house dust mite allergen extract in children and adolescents with allergic rhinitis and asthma. Immunotherapy. (2021) 13:227–39. doi: 10.2217/imt-2020-0232, PMID: 33317341

[37] Durham SR Shamji MH . Allergen immunotherapy: past, present and future. Nat Rev Immunol. (2023) 23:317–28. doi: 10.1038/s41577-022-00786-1, PMID: 36253555 PMC9575636

