# Supplementary figures and images for: A multicenter randomized study: safety of an optimized accelerated house dust mite immunotherapy for patients with allergic rhinitis in China (PERFECT study)
Source: Front Immunol. 2026 Apr 10;17:1751162. doi: 10.3389/fimmu.2026.1751162 (PMC13105971; doi:10.3389/fimmu.2026.1751162)

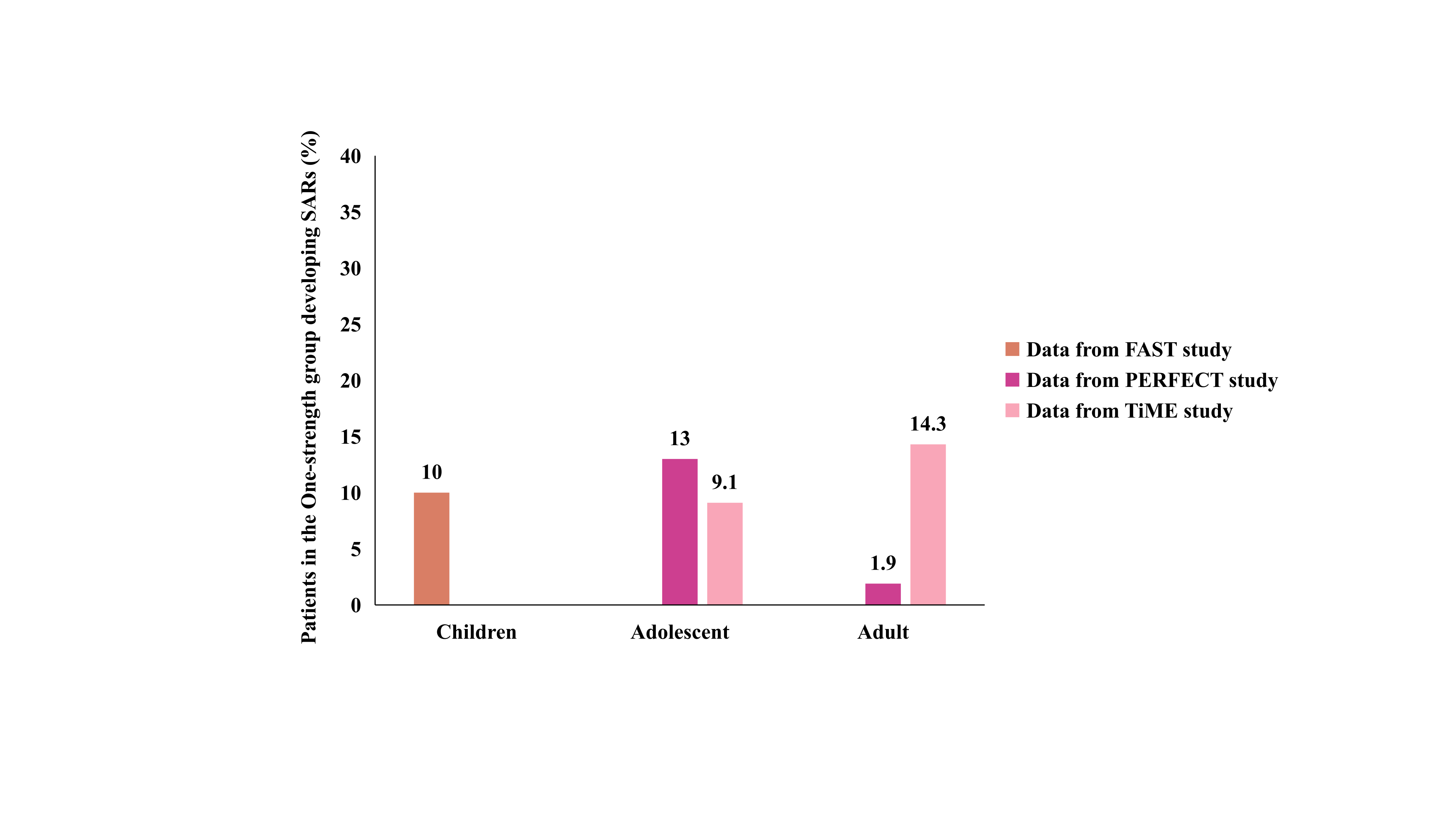

Supplement: Supplementary Figure 1 — Incidence of systemic ADRs with the one-strength scheme of the native HDM extract across FAST, TiME and this study in different age groups. ADR, adverse drug reaction. [file Image1.tiff]
