# Supplementary material for: A multicenter randomized study: safety of an optimized accelerated house dust mite immunotherapy for patients with allergic rhinitis in China (PERFECT study)
Source: Front Immunol. 2026 Apr 10;17:1751162. doi: 10.3389/fimmu.2026.1751162 (PMC13105971; doi:10.3389/fimmu.2026.1751162)
Supplement: Supplementary file 2 [file DataSheet1.docx]

**Supplementary Table 1** Number of systemic reactions occurring with strength 1, 2 and 3

|  | **One-strength group (n=108)** | | **Standard group (n=103)** | |
| --- | --- | --- | --- | --- |
|  | ***Patients, n*** | **Events, n** | ***Patients, n*** | **Events, n** |
| **Strength 1 (50 TU/mL)** |  |  | 1 | 1 |
| **Strength 2 (500 TU/mL)** |  |  | 0 | 0 |
| **Strength 3 (5000 TU/mL)** | 8 | 15 | 8 | 20 |
| **Adrenaline administration due to ADR** | 1 | 1 | 5 | 6 |

ADR, adverse drug reaction.
